# Engaging participants with research findings: A rights‐informed approach

**DOI:** 10.1111/hex.13701

**Published:** 2023-01-17

**Authors:** Mathew Sunil George, Rakhal Gaitonde, Rachel Davey, Itismita Mohanty, Penney Upton

**Affiliations:** ^1^ Health Research Institute University of Canberra Canberra Australian Capital Territory Australia; ^2^ Achuta Menon Centre for Health Science Studies SCTIMST Thiruvananthapuram Kerala India

**Keywords:** dissemination, engagement, knowledge sharing, participants, research findings, rights

## Abstract

**Background:**

Sharing research findings with participants is recognized as an ethical imperative for the research community. However, most discourse on this topic in mainstream public health takes a paternalistic approach, with researchers retaining the power to choose if, when, and how research findings are shared.

**Methods:**

Fieldwork took place from August 2018 to January 2019 and again from August 2019 to December 2019 among two communities in the south Indian state of Kerala. We integrated participant engagement with study findings into the research protocol, using various collaborative strategies identified during the design stage, forming partnerships with participants and determining appropriate forms of dissemination for different participant groups during fieldwork.

**Results:**

Findings from previous research projects undertaken with these communities by other researchers had not been shared with them. This was interpreted by the communities as researchers not being interested in making a difference to their situation. In the current study, building reciprocal relationships that minimized power disparities, and providing outputs in tailored formats that promoted active engagement were key factors that enabled participants to engage with results. This engagement added value by enabling us to co‐develop study recommendations. This process also enabled the community to have ownership of the results and use them to advocate for health system change to improve access to health care.

**Conclusion:**

Research should be transformative for participating communities. Participants have a right to know the results of the research they participate in since their knowledge provides the research data which can in turn promote community change. Operationalising this requires researchers to build partnerships with participants and their communities from the outset. The role of participants must be reimagined, and adequate resources should be built into the research process. This is both socially responsible and ethical, but also improves the impact and legitimacy of research for the participants and the communities that they represent.

**Patient or Public Contribution:**

Participants of our research contributed to the design of various aspects of the engagement processes including the venue, the formats used for engagement, interpretation of the findings and recommendations from our research.

## 1 BACKGROUND

Health research has the innate capacity to affect the lives of both the participants and the larger communities within which the research is embedded since the knowledge generated can enable societies to better organize themselves to advance their health goals.^1^ Given this significance, historically a lot of attention has been paid to ensuring scientific rigour and the integrity of processes such as research design, conduct and reporting.^2^ Viewed through this classical lens, the key stakeholders in the research process are usually the ‘expert’ researchers^3^ who plan, implement and lead all aspects of an investigation, along with institutions such as funding bodies and universities which have oversight of the research process. Research participants have a time‐limited role that ends once they provide the data that was sought from them.^4^, ^5^ However, the last two decades have seen a change in this approach and in the understanding of who the ‘expert’ is.^6^ Several theoretical frameworks, declarations and organizations have been established to change this status quo and find meaningful ways to involve participants in all stages of the research process.^4^, ^7^, ^8^, ^9^, ^10^, ^11^ Key frameworks to have emerged include the Strategy for Patient‐Oriented Research‐Patient Engagement (SPOR),^7^ the INVOLVE framework^9^ and the Patient Centre Outcomes Engagement Rubric.^11^ Whilst each framework is unique, they all share a high regard for the value of the lived experiences of research participants, an emphasis on engaging participants as equal partners across all stages of research (not just using tokenistic gestures) and a belief in the importance of sharing research findings with participants through a genuine and engaging process.

Most health research participants would like to know the results of the studies to which they have contributed.^4^, ^12^ It has been proposed that sharing study findings should be the norm, since they may have significance not just for the participant, but also for their families and communities.^13^ Evidence suggests sharing results can also increase participants' sense of ownership of research outcomes, improve trust between researchers and participants and encourage participation in future research.^14^ Studies have also shown that participants consider receiving research results as a right.^14^, ^15^, ^16^ Indeed, it has been argued that there is a moral and ethical imperative to share study findings with participants.^17^, ^18^ The Declaration of Helsinki for example states that ‘all medical research subjects should be given the option of being informed about the general outcome and results of the study’.^3^ It is also recognized that sharing study findings provides better access to participants and acceptability for the research^9^, ^11^ and dissemination of results is often included as a requirement in institutional or funding protocols.^19^ Researchers as well as funding agencies and other bodies that govern research generally support sharing results with study participants.^20^, ^21^ It is increasingly recognized that sharing results with participants should go beyond merely providing a plain language summary of key findings; it begins with engaging participants meaningfully across different stages of a research project using multiple strategies^22^, ^23^ and concludes with a shared process of discussion and interpretation of the study findings, to establish their purpose in the real word setting.

Morello‐Frosch et al.^24^ discuss three possible approaches to understanding the process of sharing research results with participants:

1. A clinical ethics framework where the focus is on reporting back individual results, especially in situations where clinical action is required to protect the participant. Here, decisions about sharing results rest with scientists and medical experts and the ownership of the data rest with the researcher(s).^25^
2. A community‐based participatory framework where there is a strong focus on communicating both aggregate and individual results to participants using protocols that are jointly developed by both the research team and the participants. Decisions about sharing results are made jointly by the researchers and the participating community.^17^ The ownership of data primarily rests with the participants from whom the data has been collected.^26^
3. Citizen‐science ‘data‐judo’ where the emphasis on reporting back both aggregate and individual results to participants is guided primarily by the policy goals that the research set out to achieve. The lead here is taken by communities and advocacy groups who have marshalled their own scientific resources to conduct a study with a view to influencing policy change.^27^

Despite this, the reality is that in many cases health research participants still do not receive study results^4^, ^28^ nor are they engaged in the feedback of findings. This is often the case with clinical trials but is also true for community‐based participatory health research.^4^ It has been argued that sharing results is not a simple task. The extent to which participants are engaged is driven by factors including the nature of the research data (the intended user, data sensitivity, ownership, target audience)^29^; the infrastructure available to facilitate data sharing^30^, ^31^, ^32^; organizational and research context (culture of the organization and discipline‐specific practices)^23^, ^33^; the individual characteristics and motivation of researcher(s) (including academic position, prior experience in sharing data as well as expected returns)^23^ and existing practices that govern research data sharing (open access publications, institutional and research repositories, sharing upon request, etc.).^34^ Open access, strongly promoted by several funding agencies and institutions,^35^, ^36^, ^37^, ^38^ while making research outputs widely available, primarily seeks to fulfil academic requirements, including establishing researchers as experts in their field. The formats of journal articles also limit the availability and useability of research findings to academic and policy audiences.

Whilst not formally rewarded by existing academic processes, the fact that this topic finds consistent mention in the literature reiterates how important it is for the research community. After all, research is a form of problem‐solving, and the value of research lies in finding answers that will ultimately benefit society—either by increasing knowledge, changing attitudes or providing practical solutions. The true benefit of research is therefore only realized once that research is shared with broader society. It is this that motivated us to develop the dissemination processes discussed in this paper. We engaged research participants with our study findings during a community‐based research project that explored access to health care for socially excluded communities in the south Indian state of Kerala. Key findings from this research have already been published,^39^, ^40^ but were also shared with the communities that participated. We involved our participants at every stage of the study in a range of processes focused on ensuring they could actively engage with project findings once initial data collection and analysis were complete. In this paper, we reflect on our experiences of engaging different participant groups, examine why a rights‐based perspective is a more effective approach to address this issue, and discuss strategies that can be adopted by researchers to improve the dissemination and application of their findings at the participant level.

## 2 METHODS

### 2.1 Study setting and participants

This study reports on the sharing of results of an ethnographic study carried out in the south Indian state of Kerala between August 2018 to January 2019 and August 2019 until December 2019.^39^, ^40^ The study sought to understand how social exclusion impacts access to health care for two marginalized communities: Indigenous communities and older widows living alone. Participants from Indigenous communities were recruited by theoretical sampling, whilst older widows living alone were identified using both theoretical and snowballing sampling strategies. The third group of participants was also recruited from the healthcare professionals (community healthcare workers, nurses and doctors) and key informants (Key informants are those who possess specialist knowledge on account of their experience, expertise and sometimes their position within a community or an organization.) connected to the two communities. These participants were recruited by using stratified purposive sampling.

### 2.2 Methodology

Participants were engaged with results during several phases of the ethnographic study: (1) Building participant rapport and trust; (2) Data collection via interviews, focus group discussions, participant observations and concurrent analysis; (3) Member checking and sharing emerging and final results with participants (the focus of this paper).

### 2.3 Dissemination planning

The decision to ensure that research findings were shared in a meaningful way with participants was made during the research design stage, with an initial dissemination plan integrated into the study protocol. Our belief that participants have the right to know the study findings were also influenced by our respect for the agency of participants. This stance evolved from our previous individual experiences as health researchers, combined with a collective appreciation for the importance of a tailored approach to meaningfully engaging participants with findings. This encouraged us to start discussions early with participants to identify which approach to sharing findings would be most effective for them. It also ensured that we set aside adequate time and resources to engage participants with findings at the end of the study.

### 2.4 Forming partnerships with participants

Ethnographic research places a great emphasis on extended contact with participants and building reciprocal relationships, as this helps avoid power disparities and gives better access to participants' lived realities. In this study, we were particularly conscious of working with socially excluded communities and the healthcare workers who provide services to these communities. Building partnerships of trust and mutual respect was therefore essential for a successful research project. This partnership‐building occurred in two stages during the research:

Once initial ideas for the research were formalised, the first author, who lived with the communities during the course of the research, held discussions with members of the population groups we expected to work with, to gauge the relevance of the topic to their lives and obtain feedback on our plans for data collection. For the Indigenous communities, meetings were held with a community‐based organisation, three Indigenous village chiefs and four healthcare workers providing services to the communities. Regarding the older widows, discussions took place with healthcare workers including two doctors and three community health workers providing services to the widows. Meetings with individual widows at this stage were not feasible due to the dispersed nature of older widows in the geographical area of research.

During the initial months of fieldwork, we took time to understand the specific contexts of different participant groups, respecting local protocols and customs during interactions, and discussing any concerns expressed about research processes and participation:

(1) Among the Indigenous communities, traditional protocols governing the interaction of outsiders with the community, such as meeting the village chief and seeking his permission, were followed.
(2) With older widows, initial interactions were facilitated by a community health worker who was a woman from the area and known to the participants. In addition to explaining key aspects of the research, the initial visit was used as an opportunity to meet the local area representative, be introduced to the neighbours and discuss suitable times and venues when data collection could happen. This was deemed necessary as communities were wary about outsiders visiting older widows living alone.
(3) Healthcare workers were met at the health facilities where they worked. The initial visit provided a forum to break the ice, engage in discussions about the relevance of the research and answer any questions.

Such extended and multi‐layered contact enabled the first author to become familiar with the participants and move from being the ‘outsider expert’ to an ‘accepted’ member of the group. This also enabled the first author to better understand existing power differentials such as those between the health system and community participants, as well as between the first author as an expert outsider and study participants. This supported the first author to build relationships that were reciprocal and respectful.

### 2.5 Tailoring dissemination

Having different groups of participants required us to evolve appropriate mechanisms that promoted active engagement and sharing of research findings with each group. This involved negotiating the appropriate location and arrangement for disseminating study findings.

The findings were also shared with a community‐based advocacy organization in Attapadi, Kerala. This was to ensure that dissemination of study findings extended beyond immediate participants to relevant community‐based groups working in local Indigenous health and wellbeing. We were unable to identify a suitable organization to disseminate findings related to older widows living in the community, as most community‐based groups working with older widows catered primarily for those in residential care.

## 3 FINDINGS

### 3.1 Legacy of nondissemination

During the initial phase of fieldwork, the impact of previous research findings not being shared was a key barrier to building rapport. Several research groups had carried out data collection with the Indigenous communities, but hardly anyone returned to share findings. This lack of engagement by researchers post‐data collection, made community members feel researchers were far more interested in ‘getting their work done’ rather than helping them address their situation. This feeling was echoed by other participant groups, including older widows. Some even wondered if researchers accurately reported the information that they had shared in their outputs. They felt that this was unfair as they had spent time and shared information about their lives with researchers. Such experiences also meant that communities were reticent about participating in further research as they considered such exercises futile.

People like you come here for research, but once they finish their work, there is no sign of them. They don't tell us what they have found out and neither do we know what has happened to the report that they wrote. Once they have collected whatever they want no one ever comes back. (Indigenous Community FGD, TI10)

Health system participants (especially doctors) pointed out the benefits of disseminating the results locally and the limitations of not doing so. Many felt that research could only initiate change in the health system where it was carried out if researchers shared their findings in a locally meaningful way.

I don't think so far anyone has come and spoken about their findings here. Usually what happens is that people will do some surveys or carry out interviews. But once that is over, they do not come back. We won't even know where they are or what happened to them after that. (Healthcare provider IDI, MO_4)

### 3.2 Inaccessible research outputs

Most research findings from previous studies conducted in these communities were available as official reports and/or journal articles. However, accessing and making sense of their contents required resources such as internet access and specific skills including proficiency in written English and an understanding of health research terminology. These were significant barriers for community participants (Indigenous people and widows), who were unable to read reports or documents from previous research. Healthcare professionals were also unaware of where to find the outputs of previous research and how to apply the findings to their practice. What evolved in our consultations was that a verbal presentation followed by a discussion of the results either in an individual or a group setting would be far more effective for all participant groups. Merely receiving a copy of a research report was not going to help them engage with the findings or apply them to their lives.

I don't know what they are writing about us. I hope they are writing what we say (laughs). But what can we do about it? We can't read what they write, and they also don't tell us what they finally write in those reports. (Indigenous community FGD, TK12)

### 3.3 Familiar forums foster engagement

Among the Indigenous communities, the village assembly presided over by the village chief was chosen as the appropriate forum to share findings. Aggregate findings were presented one by one followed by a discussion about the key issues. Using this traditional forum and discussion style provided the Indigenous communities with a familiar context which enabled greater engagement and empowered participants to comment on the findings and correct our interpretation.

With the widows, individual meetings were held to talk about the findings on a one‐to‐one basis in their homes. This suited them better as these participants lived by themselves. Gathering them together would have disrupted their daily routine and put them in the company of others they did not know well. This would have diminished their ability to engage with the findings, ask questions and comment on them freely.

Among healthcare providers, one‐to‐one discussions were favoured. As with community participants, aggregate findings were shared followed by a discussion and comments on the findings. This allowed participants to discuss findings in some detail, clarify our interpretation and provide further input. Some participants preferred to learn about the findings at the health facility, while others asked for the discussion to be held away from their place of work.

### 3.4 Contextualizing findings increase understanding

Our discussions with the different groups of participants also established that different presentation formats would be more effective for each group. Before presenting study findings, discussions were held with a small number of participants to explore the best format to present the results. These discussions helped us to understand the importance of using imagery, language and contextual information relevant to each population group instead of a generic approach. For example, while discussing the gradual decline of Indigenous healing traditions and the increasing acceptance of western medicine, parallels were drawn to how the official language of the state (Malayalam) had taken over tribal dialects, resulting in tribal terms being replaced with Malayalam words. Thus, the Indigenous word for mother ‘avva’ was replaced by the Malayalam word ‘amma’. Using this language resonated with the community and led to discussions about how the neglect of healing traditions should be seen within the overall context of Indigenous ways of life (including language) not being valued. Among the widows, the key findings were presented within the context of the altered family structure that the state of Kerala had witnessed in the last two decades where the social understanding of what constituted a ‘family unit’ and responsibilities for caring for older family members had changed considerably. This resonated with the lived context of our research participants and set the tone for engaging discussions as they were able to relate our findings to their lives. Likewise, healthcare providers were able to engage better with the findings when they were presented within the context of their experiences of providing health care.

### 3.5 Two‐way communication adds value

Two‐way communication that occurred as a result of the various processes adopted during the dissemination of findings added value to the research in two specific ways. First, it confirmed that the results captured the lived reality of the participants. Second, this process facilitated the co‐construction of findings and enabled us to jointly evolve recommendations from the study. One finding, for example, was about the importance of decentralizing the delivery of healthcare services. During our discussions, some of the widows pointed out that there was a specific aspect of decentralization that would help them the most. Participants explained that while the neighbourhood clinics had made a difference in their ability to access healthcare services, they still had to travel great distances for diagnostics. This feedback enabled us to evolve a specific recommendation about decentralization that was relevant to the community.

### 3.6 Handling multiple perspectives

Some of the study findings showed that the planning and delivery of healthcare services made access difficult for communities. In the case of Indigenous communities, the centralization of health care was a decision taken to ensure that most services were provided under the supervision of specialist doctors. However, this had the impact of excluding many of the villagers who lived far away from this hospital. With the widows, the issue of physical infrastructure that was not friendly for older adults was highlighted as a deterrent to visiting public healthcare facilities even when care was required. We had anticipated that some of the health system participants, particularly those in charge of the local health system might be unhappy with findings that appeared critical of their service provision. We addressed this by ensuring that the language used while describing results did not suggest that any specific individual or institution was responsible for any negative findings. Thus, while discussing the issue of centralization, we encouraged participants—particularly those from the health system—who disagreed with the findings, to present their side of the story. Second, we were careful to communicate to all participants that our findings were meant to identify systemic issues rather than imply criticism of one individual or institution.

The group discussion format employed to engage Indigenous participants enabled greater engagement and discussion among all those present. However, it also posed some unique challenges. For example, in one of the early village meetings, a community member objected to findings that he felt portrayed the local health system in a poor light. We prepared for this by clearly communicating at the beginning of each meeting that it was acceptable to disagree with the results that were being shared and that everyone present had a right to discuss the results and disagree or comment on them.

### 3.7 Using results to advocate for change

An important, though unplanned, the outcome of the process used to share findings with participants was how they were able to own the results and use them to advocate for change. The findings from our study were used as part of the Indigenous manifesto that was released before the legislative assembly elections in the state of Kerala in 2021. This manifesto was shared with the major political parties in the state of Kerala thereby promoting visibility and debate on the study findings. Over the last 2 years, the results were also used to lobby and advocate for decentralization and primary health care in Attapadi and the appointment of 41 Indigenous young women as front‐line community healthcare workers. This was made possible because the research findings were:

1. discussed and shared in a manner that made them accessible to participants and the wider community to which they belonged, promoting ownership of study results;
2. shared with a community‐based organization working among the Indigenous communities which was involved in health advocacy. This organization was able to use the research findings to drive policy change.

This also demonstrated the importance of sharing results and engaging key stakeholders such as community‐based organizations as allies to drive change and impact.

## 4 DISCUSSION

The last three decades have seen a growing interest in engaging research participants and ensuring that the findings of health research are freely available to those beyond the traditional consumers of research outputs. Most discussions about sharing research findings state that participants would like to know about the findings.^12^, ^15^, ^41^ However, it is also acknowledged that in practice research findings are not shared as often as they should be.^15^, ^22^, ^41^ Indeed, whilst health researchers agree on the importance of making research publicly accessible, it is often admitted that it is ‘easy to become untethered from these foundational principles’.^42^ The failure to share research findings with participants has been shown to impact researcher credibility and an individual's future participation in research.^4^, ^43^, ^44^ We found this to be true in our study. Most participants had taken part in research previously, and not knowing what was written about them made them feel that research and researchers were more concerned about their own careers than improving community living conditions.

**Figure 1:**
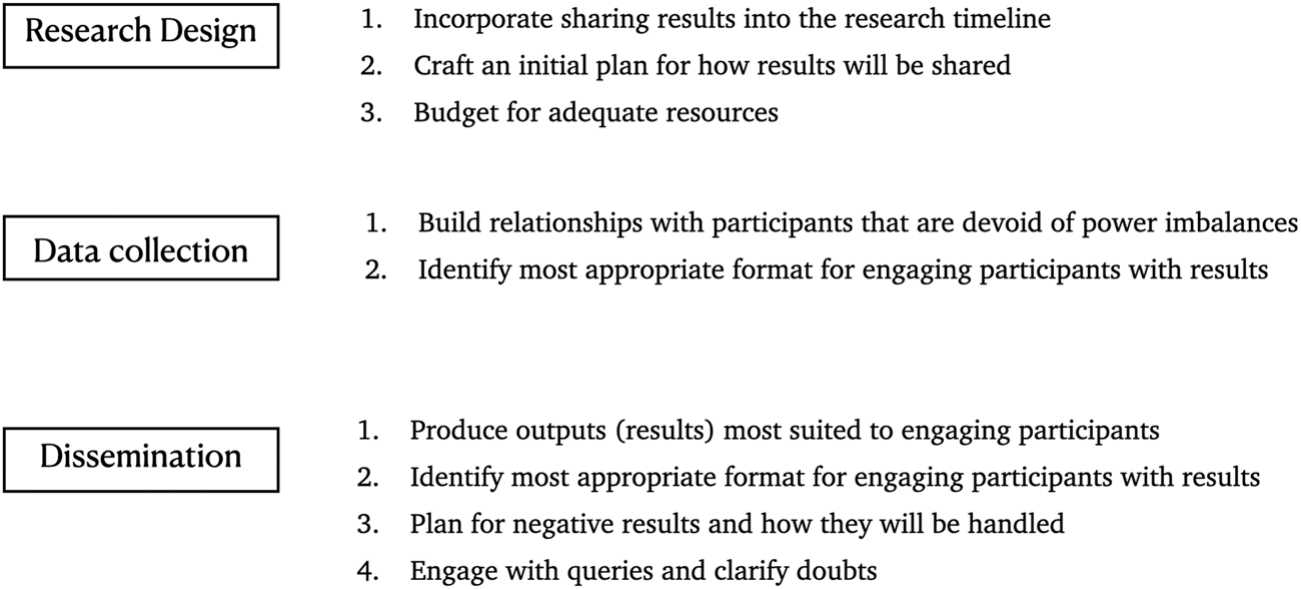
Activities to engage participants with results in different research phases

Publishing in open access journals is highly recommended as a way of making research results widely available. However, journal publications are unsuited to the effective dissemination of results to research participants and in fact, heighten the power difference between local communities and researchers.^45^, ^46^ A lot of research published about the Indigenous communities who took part in this study was available online in the form of reports, journal articles and newspaper reports. However, for participants to access these, understand the key issues and relate this to their lives, requires a specific set of skills including fluency in English, access to the internet, as well as knowledge of technical terminology common in such outputs. Not having these skills is a key barrier to participants' access to research findings. A more effective mechanism for dissemination to participants is to arrive at formats of sharing via negotiated consensus. These formats should take account of participant profiles including language spoken at home, knowledge‐sharing traditions (oral vs. written), local context and culture. Doing this enabled us to arrive at three different formats of sharing study findings which helped participants to engage with findings effectively. For this to occur, the value of sharing research findings with participants must be incorporated into the research design, and steps should be taken throughout the research process to engage with participants and identify the most effective formats for them to engage with the findings (Figure 1). While the actual sharing of results occurred towards the end of the study, engaging with participants throughout the research process was critical in ensuring that we moved beyond the tokenistic sharing of results to active participant engagement. The various engagement activities from forming partnerships to tailoring dissemination and promoting two‐way communication ensured that our participants had control over some of the decisions that were taken regarding the sharing of results such as the format, the location and the forums that were used. These processes ensured that participants moved from nonparticipation or tokenism to involvement at the level of citizen power as described by Arnstein while discussing citizen participation.^47^ Achieving this from our experience requires that the sharing of results needs to be planned early and is in keeping with previous recommendations.^2^

While the literature provides several reasons for research findings not being shared with participants, one issue that gets little attention is the fact that current reward structures for researchers rarely incentivize the sharing of research findings beyond publications in high‐impact journals.^22^, ^28^ This is unlikely to change until the role of the academic is redefined to enable researchers to move beyond theory development and testing to embrace research as a strategy for social change. There is a movement within academia to change this approach with various frameworks (e.g., Participatory Action Research [PAR], transformative research approaches and feminist methodologies) espousing the value of working with study participants as equal partners throughout the research process, including sharing study findings. However, a lack of validation at a systemic level makes this a challenge, one which can only be overcome with a change in how we (mainstream public health academics) conceptualize and teach about the role of health research, the values that we instil in research students, and the criteria by which academic success is judged. Cross‐disciplinary insights particularly from fields focused on social causes such as sociology and social work could also be of benefit.

One question that researchers might have about sharing findings is, what will participants and their communities do with the findings that have been shared with them? We found that one of the important outcomes of the process we followed, was the co‐construction of study findings which gave the community ownership. This in turn enabled the community to use them to drive change in the local health system and raise some of the issues identified in larger debates about access to health care with government officials. The involvement of a community‐based advocacy group, which also participated in the research, acted as a catalyst in this process. This clearly shows that communities have agency and when research findings are shared with them in the right format and committed community organizations are included in the dissemination process, they can use that information to take positive action and drive locally relevant change. This process of co‐construction by the participants and their communities further helped to address the power differentials that are a norm between researchers and study participants, by giving them the opportunity to decide how they are defined^48^ and providing appropriate input to the study findings. This agreed interpretation of study data ensured that the findings were not only transparent, accessible and relevant to participants' lives, but more importantly, it strengthened the validity of the findings, ensuring they were an accurate reflection of participant values, beliefs and experiences.

Communicating effectively with participants about study findings requires researchers to work with participants to identify the most suitable way to do so.^49^ In our experience, this is key if participants are to fully engage with results. Attending the village assembly for the Indigenous community participants, and one‐to‐one meetings at a location chosen by the older widows and health system participants, enabled greater engagement, as opposed to a one size fits all approach. Avoiding technical jargon and using local idioms, images and phrases where feasible is also essential. This may well be an area where researchers need training in forms of communication that are effective when communicating with lay audiences.

### 4.1 Rights‐based approach to engaging participants with results: A way forward

Research participants make an important contribution to the success of any research by sharing their information. Without this contribution, research is not possible. This we believe grants participants the right to know the outcomes of the research they were involved in. Most discussions on engaging participants and sharing research results promote this either as an ethical stance that the researcher should take or as a way to add value to the research.^4^, ^7^, ^8^, ^9^, ^10^, ^11^, ^24^, ^25^ A rights‐informed approach goes beyond these by perspectives not only including in its ambit ethical and accountability arguments but also acknowledging that participants, by virtue of their contributions to the success of the research, have a right to know the outcomes. Second, it frames the sharing of results as an obligation to participants, rather than an optional activity that can be left to the discretion of researchers or institutions. This also addresses the power differentials that other approaches confer on researchers and institutions when it comes to sharing results.

As previously noted, transformative research frameworks such as PAR already promote sharing research findings as an effective strategy for addressing the power imbalance between participants and researchers.^26^, ^50^ We believe, however, that sharing findings with participants should not be restricted to frameworks such as PAR but rather is relevant to all health research. It is the right of every research participant, not only those who participate in community‐based research.

When research participants do not have access to the results, it further adds to the distance between researchers and participants. Consequently, research remains something that is distant and disconnected from people's daily lives and their improvement.^51^ Engaging participants with results is an effective way to address this and makes the benefits to participants and their communities far more tangible. It also helps to ensure that research participants welcome and value research as a means to solving issues pertinent to their lives thereby encouraging the participation of individuals and communities.

Recognizing, respecting and valuing the rights, agency and ability of research participants to engage in the dissemination process should not be an optional activity. It is essential for bringing together the key research processes of investigation, identification and implementation of ideas. It bridges the gap between the discovery and delivery of new knowledge. A rights‐informed approach to engaging participants with study findings is not only socially responsible and an ethical imperative, but it also has implications for the influence and impact of research where it most counts—study participants and the population they represent.

## AUTHORS CONTRIBUTIONS

Mathew Sunil George carried out the data collection and drafted the manuscript with contributions from Rakhal Gaitonde, Rachel Davey, Itismita Mohanty and Penney Upton. Mathew Sunil George, Rakhal Gaitonde and Penney Upton were involved in the analysis of the qualitative data. All authors were involved in the design of the larger study from which the data for this manuscript emerged. The authors read and approved the final manuscript.

## CONFLICT OF INTEREST

The authors declare no conflict of interest.

## ETHICS STATEMENT

The Human Research Ethics Committees of the University of Canberra (20180074) and the Indian Institute of Public Health Delhi (IIPHD_IEC_03_2018 provided ethical approval for this study. Regulatory permissions were obtained from the Kerala Department of Health (GO(Rt)No2677/2018/H&FWD) and the local administration in Attapadi. Permissions were also obtained from the local health officials before fieldwork began. All participants gave informed consent before data collection.

## Data Availability

The data sets generated and/or analysed during the current study are not publicly available as they are qualitative in nature but are available from the corresponding author upon reasonable request.
